# The Impact of Endoscopic Linear Stapling Device Stability in Thoracic Surgery: A Delphi Panel Approach

**DOI:** 10.36469/9843

**Published:** 2016-10-28

**Authors:** Daniel Miller, Diego Gonzalez Rivas, Kellie L. Meyer, Ryan S. Clark, Tadasu Kohno

**Affiliations:** 1 Professor of Surgery, Wellstar Health System, Marietta, GA, USA; 2 Head of Minimally Invasive Thoracic Surgery, Coruña University Hospital, Coruña, Spain; 3 Senior Director, Xcenda, Palm Harbor, FL, USA; 4 Manager, Xcenda, Palm Harbor, FL, USA; 5 Chief of Thoracic Surgery, Toranomon Hospital, Tokyo, Japan

**Keywords:** delphi panel, video-assisted thoracic surgery (vats), stability, endoscopic stapling device), thoracic surgery

## Abstract

**Objectives:** To develop consensus statements outlining the impact of endoscopic linear stapling device stability on potential complications of thoracic surgery and the stress/concern of thoracic surgeons.

**Methods:** Eight thoracic surgeons representing 8 countries participated in a Delphi panel process using 2 anonymous surveys. The first included binary, multiple-response, and Likert scale-type questions, which were converted into affirmative statements for survey 2 if an adequate number of respondents answered similarly. Consensus was defined a priori when ≥70% agreed with the affirmative statement in survey 2.

**Results:** All panelists completed both surveys. Panelists unanimously agreed that: 1) an endoscopic linear stapling device with improved stability would result in less stress/concern for critical firings, surgeries where a fellow is trained, and robot-assisted surgeries requiring an assistant; 2) reduced unintentional tissue/structure damage and reduced tension on tissue being fired upon may result from use of an endoscopic linear stapling device that provides improvement in stability; and 3) endoscopic linear stapling device stability had more clinical importance in video-assisted thoracic surgery compared to open thoracic surgery.

**Conclusions:** Improved endoscopic linear stapling device stability is a critical component of thoracic surgery likely to result in more frequent positive surgical outcomes when compared to a device with greater instability.

## INTRODUCTION

Lung surgery that resects a portion of the lung may be indicated for a number of thoracic diseases, such as lung cancer or severe emphysema.[1] In 2011, approximately 250,000 patients in the United States underwent a lung operation, with an average hospital length of stay of 8.1 days and cost of $20,349.[2] Furthermore, the incidence of lung cancer is rapidly growing in parts of Asia, making more patients eligible for lung resection surgery in the future.[3] More recently, video-assisted thoracic surgery (VATS) has enabled surgeons to perform thoracic operations with smaller incisions and improved visualization, leading to better surgical outcomes over thoracotomy, such as a decreased pain, post-operative complications (e.g. prolonged air leaks) and length of hospital stay.[4,5,6]

Endoscopic linear stapling devices have enabled the initiation and quick evolution of VATS lung resections by reapproximating tissue with decreased parenchyma bleeding during these minimally invasive thoracic procedures.[7] However, movement of the endoscopic device during stapling may lead to instability of the distal tip, which may increase the incidence of complications during and after thoracic surgical procedures. More recently developed devices have minimized inadvertent movement at the distal tip of the device, ensuring optimal control and protection when working on critical structures. These newer devices provide at least 60% less movement at the end effector during transection of thick tissue.[8]

The objective of this study was to develop consensus statements outlining the impact of endoscopic linear stapling device stability on potential complications of thoracic surgery and the stress and/or concern of thoracic surgeons related to endoscopic linear stapling device stability using an electronic Delphi panel approach.

## METHODS

A Delphi panel process was employed for this study, which uses a series of questionnaire rounds to gather information until professional consensus is reached. The Delphi technique is often used to ascertain and categorize evidence established from group involvement or a panel of experts. This method is especially beneficial as many individuals across diverse settings can anonymously provide input, which avoids domination of the consensus process by one or a few experts and allows participants to respond more openly.[9,10] A Delphi panel was used for this study because the relationship between device stability and surgical outcomes could not be assessed by retrospective analyses, as no mechanism is in place to capture the movement of a surgical device during thoracic procedures. Whilst prospective studies are a potential approach, the sample size required to demonstrate a causal relationship would be prohibitive and results would be inconclusive due to the multi-factorial nature of post-operative complications.

Delphi studies aim to recruit individuals who, based on criteria such as professional experience, education, or employment, are perceived to have expertise in the subject matter being studied.[11] An eight-member panel of practicing thoracic surgeons was self-selected based on willingness to complete the surveys. The group was geographically diverse, representing eight different countries. All surgeons significant surgical experience and were familiar with and experts in Video Assisted Thoracoscopic Surgery (VATS) approach for pulmonary resections using laparoscopic stapling devices. Additional panel demographics can be found in Table 1.

The protocol for this study included two (2) rounds of web-based surveys (web survey tool: ConfirmIt). The first round of the survey included binary, multiple-response, and Likert scale type questions, and the second round of the survey included only binary questions in response to affirmative statements. Although individuals who provided the responses remained anonymous, aggregate results from survey 1 were provided to panelists completing survey 2 to facilitate consensus. Binary questions (eg, yes/no) were converted to affirmative statements in survey 2 if ≥25% of respondents selected a positive response in survey 1. "Check all that apply" questions were converted to affirmative statements in survey 2 if ≥25% of respondents selected a particular response in survey 1. Questions rated on a 5-point Likert scale were converted to an affirmative statement in survey 2 if the response average was >2.5 or all survey participants responded with a 3 or higher in survey 1. Those questions in survey 1 that were not converted to an affirmative statement were considered unlikely to reach consensus and removed (included in the APPENDIX). Consensus opinion was considered *a priori* when ≥70% of respondents agreed with the affirmative statement in survey 2.

**Table 1.** Advisor Characteristics

| Characteristic | Value |
|---|---|
| ***n*** | 8 |
| **Average years of experience in thoracic surgery (range)** | 13.4 years (6 - 23 years) |
| **Average thoracic procedures per month (range)** | 38.8 (20 - 75) |
| **Practice setting** | |
| Private practice | 7 (87.5%) |
| Large academic teaching hospital | 1 (12.5%) |
| **Perform robot-assisted procedures** | |
| Yes | 2 (25%) |
| No | 6 (75%) |
| **Countries represented** | Denmark<br>Germany<br>Hong Kong<br>Italy<br>Japan<br>The Netherlands<br>United Kingdom<br>United States |

"Device stability" or "improved" stability" was defined as decreased movement of the distal tip/end of the endoscopic linear stapling device. Panelists were not provided any publications or information related to device instability in thoracic surgery and, as a result, any expert consensus achieved in this study was determined solely by the clinical experience of the expert panel. Questions from the following categories were posed to panel members in surveys 1 and 2: Impact of instability, impact of device stability on surgeon stress/concern, impact of device stability on specific tissue types, device stability in VATS and open thoracic surgery, clinical importance of device stability and improved device stability. A detailed list of the survey 1 and survey 2 questions appears in the Appendix.

## RESULTS

All 8 panelists (100%) completed survey 1 and survey 2. Of the 105 potential statements tested in survey 1, 98 (93%) were converted to affirmative statements and tested in survey 2.

## Impact of Instability

When firing an endoscopic linear stapling device, a number of negative outcomes may occur due to movement of the distal tip/end (vertically, horizontally, or laterally). In addition to increased tension on the structure being fired upon, the panel agreed that unintentional tissue damage could occur due to movement during firing. The panel also achieved consensus on the statement that tearing of fragile tissue, either away or along the staple line, is also a significant concern with tip instability while articulating. Poor staple line quality was not considered to be a potential negative outcome of distal tip movement when triggering the device by the majority of panelists.

Panelists unanimously agreed that lung trauma causing an air leak and unintentional tissue and/or structure damage could result from too much movement of the distal tip or end of the endoscopic linear stapling device. Additionally, staple line oozing or bleeding controllable with clips, suture or fibrin glue, and tearing of fragile tissue along and away from staple line were considered potential hazards of tip instability. Although half of the panelists reported observing staple line oozing that required additional OR time, bleeding, bronchus trauma, tension on the structure being fired on, and an increased likelihood of damaging a non-visible structure to be a concern with excessive tip movement, consensus was not reached.

Detailed results of the impact of instability on surgical outcomes can be found in Table 2.

**Table 2.** Survey 2 Results on the Impact of Instability

| Statement | Affirmative Responses n (%) |
|---|---|
| **Complications that could occur with movement of the distal tip/end when firing the device** | |
| Unintentional tissue damage | 8 (100%)* |
| Tearing of fragile tissue along the staple line | 8 (100%)* |
| Increased tension on the structure or tissue I am firing on | 7 (88%)* |
| Tearing of fragile tissue away from the staple line | 6 (75%)* |
| Poor staple line quality | 3 (38%) |
| **Complications that could occur with too much movement of the distal tip/end** | |
| Lung trauma that causes an air leak | 8 (100%)* |
| Unintentional tissue / structure damage | 8 (100%)* |
| Staple line oozing / bleeding controllable with clips, suture or fibrin glue | 7 (88%)* |
| Tearing of fragile tissue along staple line | 7 (88%)* |
| Tearing of fragile tissue away from the staple line | 6 (75%)* |
| Staple line oozing that requires 15 minutes or more of additional OR time | 5 (63%) |
| Bleeding that requires conversion to open | 4 (50%) |
| Bleeding that requires blood transfusion | 4 (50%) |
| Bronchus trauma that requires further repair | 4 (50%) |
| Likelihood of damaging an important structure I am unable to see while firing | 4 (50%) |
| Tension on the structure or tissue I am firing on | 4 (50%) |

*Consensus achieved

## Impact of device stability on surgeon stress and/or concern

Thoracic surgeons must use a number of tactics to help mitigate the potential complications that may result due to a lack of endoscopic linear stapling device stability during surgery. Unanimously, panel members required use of a second hand to stabilize the device at the site of entry and took additional care to staple slowly and stably (in general and during critical firings). A firm / tight grip was also needed during articulation to prevent movement.

The involvement of other individuals in the OR can cause a high level of stress and concern for thoracic surgeon. Panelists agreed that surgical training can be stressful for the trainer due to concern regarding the surgeon-in-training's* ability to minimize movement. Furthermore, during robot-assisted thoracic surgery, both panelists who perform this type of surgery indicated experiencing instability-related stress when an assistant fires an endoscopic linear device.

As the potential for device distal tip movement is considered to be worrying, it is unsurprising that the panel unanimously agreed a linear stapling device with improved stability would result in less stress and concern. This result held consistent for critical firings, surgical procedures where a fellow was being trained, and robot-assisted surgeries requiring an assistant. Detailed results of the impact of device stability on surgeon stress and/ or concern can be found in Table 3.

## Impact of device stability on specific tissue types

The outcomes benefit provided by improved stability of an endoscopic linear stapling device differs by tissue type. In this study, five distinct surgery targets were tested – thin, critical structures (such as pulmonary artery / pulmonary vein); thick, less critical tissue (such as lung parenchyma); bronchus tissue; lung tissue of poor quality (such as emphysematous tissue, tissue damaged due to radiation, chemotherapy, or inflammation); and tissue that requires working in tight spaces with poor visualization. Across all tissue types, panelists unanimously agreed that reduced unintentional tissue / structure damage and reduced tension on the tissue being fired upon may result from the use of a device that provides an incremental improvement in stability. Additionally, better stability was considered a positive factor in all tissue types except for bronchus tissue for the following complications: reduced tearing of fragile tissue along the staple line, reduced staple line oozing / bleeding, and reduced lung trauma that leads to fewer / less severe air leaks. Conversely, consensus was not reached on whether improved device stability reduced the likelihood of damaging an important structure unable to be seen, reduced the bronchus trauma, or improved the staple line formation for any tissue type. Based on the number of tested negative outcomes avoided, lung tissue of poor quality received the most benefit from an improvement in stability whereas bronchus tissue was relatively unaffected by tip stability.

Overall, the number of positive outcomes associated with improved device stability in thin critical structures and thick, less critical tissues reaching consensus were similar. Per panel consensus, an incremental improvement in device stability for both tissue types may result in reduced unintentional tissue damage, reduced tearing of fragile tissue along the staple line, reduced tension on the tissue being fired upon, reduced staple line oozing / bleeding, reduced bleeding resulting from tissue damage, and reduced lung trauma leading to fewer / less severe air leaks. Unlike thick tissue, improving tip stability in thin tissue may result in decreased trauma to surrounding tissue / structures.

*Note: surgeon in training may refer to an intern or fellow. The question was asked as, "When training a fellow during thoracic surgery, I am stressed / concerned with their ability to minimize movement"

**Table 3.** Survey 2: Results on theImpact of Device Stability on Surgeon Stress/Concern

| Statement | Affirmative Responses n (%) |
|---|---|
| **Actions to mitigate potential complications related to a lack of endoscopic linear stapling device stability** | |
| Stabilize the device with a second hand at the site of entry | 8 (100%)* |
| Take additional care to staple slowly and stably | 8 (100%)* |
| Take extra care during critical firings to minimize movement | 8 (100%)* |
| Hold the stapler device more firmly / tightly when firing | 7 (88%)* |
| Ask an assistant for aid in stabilizing the stapler | 4 (50%) |
| **Thoracic surgeon stress / concern experienced during surgery** | |
| When training a fellow during thoracic surgery, I am stressed / concerned with their ability to minimize movement | 8 (100%)* |
| I experience stress/concern when an assistant fires an endoscopic linear stapling device during a robot assisted thoracic surgery procedure | 2 (100%)† |
| **Impact of an endoscopic linear stapling device on stress / concern** | |
| I anticipate I would have less stress/concern if I were using an endoscopic linear stapling device with improved stability during critical firings | 8 (100%)* |
| I anticipate I would have less stress/concern if I were training a fellow during thoracic surgery with an endoscopic linear stapling device that has improved stability. | 8 (100%)* |
| I anticipate I would have less stress/concern when an assistant is firing an endoscopic linear stapling device with improved stability during a robot assisted thoracic surgery procedure. | 2 (100%)† |

*Consensus achieved; †2 panel members perform robot-assisted procedures

According to the study panel, incremental improvement in tip stability does not result in positive clinical outcomes when operating on bronchus tissue – in fact, the panel only agreed that reduced unintentional tissue damage and reduced tension on the structure being fired upon may result. Lung tissue of poor quality is very different in that all but three positive outcomes tested reached consensus. Those outcomes that did not reach consensus (reduced likelihood of damaging an important structure unable to be seen, reduced bronchus trauma, and improved staple formation) did have 50% (4) of panelists perceive incremental stability potentially having an impact.

Finally, when examining operating in tight spaces where visualization of surrounding structures is poor, improved stability may result in reduced unintentional tissue / structure damage, reduced tearing of fragile tissue along the staple line, reduced tension on the structure being fired upon, reduced lung trauma, and decreased trauma to surrounding tissue. Detailed results of the impact of device stability on specific tissue types can be found in Table 4.

**Table 4.** Impact of Device Stability on Specific Tissue Types

| | Affirmative Responses for Thoracic Surgery that Targets [n (%)]: | | | | |
|---|---|---|---|---|---|
| **Response** | **Thin critical structures** | **Thick, less critical structures** | **Bronchus tissue** | **Lung tissue of poor quality** | **Tight spaces** |
| Reduced unintentional tissue / structure damage | 8 (100%)* | 8 (100%)* | 6 (75%)* | 8 (100%)* | 6 (75%)* |
| Reduced tearing of fragile tissue away from the staple line | 5 (63%) | 5 (63%) | 3 (38%) | 7 (88%)* | 5 (63%) |
| Reduced tearing of fragile tissue along staple line | 7 (88%)* | 8 (100%)* | 4 (50%) | 8 (100%)* | 6 (75%)* |
| Reduced likelihood of damaging an important structure I am unable to see while firing | 4 (50%) | 4 (50%) | 4 (50%) | 4 (50%) | 5 (63%) |
| Reduced tension on the structure or tissue I am firing on | 6 (75%)* | 8 (100%)* | 6 (75%)* | 7 (88%)* | 6 (75%)* |
| Reduced staple line oozing / bleeding | 7 (88%)* | 7 (88%)* | NA | 7 (88%)* | 6 (75%)* |
| Reduced bleeding resulting from reduced damage to tissue / structure | 8 (100%)* | 6 (75%)* | NA | 6 (75%)* | 5 (63%) |
| Reduced lung trauma, resulting in fewer / less severe air leaks | 7 (88%)* | 7 (88%)* | 3 (38%) | 8 (100%)* | 6 (75%)* |
| Reduced bronchus trauma | 4 (50%) | NA | 5 (63%) | 4 (50%) | 4 (50%) |
| Improved staple formation | 5 (63%) | 3 (38%) | 5 (63%) | 4 (50%) | 5 (63%) |
| Improved staple line quality / integrity | 5 (63%) | 3 (38%) | 5 (63%) | 6 (75%)* | 5 (63%) |
| Decreased trauma to the surrounding tissue / structures | 5 (63%) | 8 (100%)* | NA | 6 (75%)* | 6 (75%)* |

*Consensus achieved

*Device stability in VATS and open thoracic surgery*

The panel unanimously considered endoscopic linear stapling device stability to have more clinical importance in VATS thoracic surgery compared to open thoracic surgery. Although the reasons for this vary, most panelists cited the limited space and more critical application of VATS compared to the more robust set of instruments and support available with open surgery. This study examined several issues related to VATS thoracic surgery that would be less stressful / concerning when performing a surgery with an endoscopic linear stapling device that has improved stability. According to panel consensus, a more stable tip can mitigate the stress and concern of space restrictions, having to work adjacent to critical structures, and inability to take corrective action. Detailed results of device stability in VATS and open thoracic surgery can be found in Table 5.

**Table 5.** Survey 2: Results on Device Stability in VATS and Open Thoracic Surgery

| Statement | Affirmative Responses n (%) |
|---|---|
| **Endoscopic linear stapling device stability has more clinical importance in VATS thoracic surgery compared to open thoracic surgery.** | 8 (100%)* |
| **Potential Issues related to VATS that would be less stressful/concerning with improved tip stability** | |
| Having to work adjacent to critical structures | 7 (88%)* |
| Inability to take corrective action | 6 (75%)* |
| Space restrictions | 6 (75%)* |
| Difficult angles | 5 (63%) |
| Decreased visibility | 2 (25%) |

*Consensus achieved

*Clinical importance of device stability and improved device stability*

This study found consensus among panelists that when considering thoracic surgery in general, endoscopic linear stapling device stability is clinically very important. Having a stable tip is even more important in VATS, where stability is considered extremely clinically important. Despite the higher level of importance in VATS, a steady stapling device is unanimously considered to be clinically important.

All panelists agreed that, in general, an improvement in endoscopic linear stapling device stability in thoracic surgery would have a positive clinical effect on surgical outcomes. When specific outcomes were considered, panelists unanimously agreed that reduced unintentional tissue / structure damage, reduced tearing of fragile tissue along the staple line, reduced lung trauma, and reduced staple line oozing / bleeding may occur with improved stapling device stability. Among other outcomes that reached consensus, reduced bleeding resulting from reduced damage to tissue and reduced tension on the tissue being fired upon were also considered to be positively impacted by a more stable stapler device. Detailed results of the clinical importance of device stability and improved device stability can be found in Table 6.

**Table 6.** Survey 2: Results on the Clinical Importance of Device Stability and Improved Device Stability

| Statement | Affirmative Responses n (%) |
|---|---|
| **Endoscopic linear stapling device stability and associated clinical importance** | |
| Open thoracic surgery (clinically important) | 8 (100%)* |
| General thoracic surgery (very clinically important) | 7 (88%)* |
| VATS (extremely clinically important) | 7 (88%)* |
| In general, an improvement in endoscopic linear stapling device stability in thoracic surgery would have a positive clinical effect on surgical outcomes | 8 (100%)* |
| **Specific positively impacted outcomes with improved device stability** | |
| Reduced unintentional tissue / structure damage | 8 (100%)* |
| Reduced tearing of fragile tissue along staple line | 8 (100%)* |
| Reduced lung trauma, resulting in fewer / less severe air leaks | 8 (100%)* |
| Reduced staple line oozing / bleeding | 8 (100%)* |
| Reduced bleeding resulting from reduced damage to tissue / structure | 7 (88%)* |
| Reduced tension on the structure or tissue I am firing on | 6 (75%)* |
| Improved staple line quality / integrity | 5 (63%) |
| Decreased trauma to the surrounding tissue / structures | 5 (63%) |
| Reduced tearing of fragile tissue away from the staple line | 5 (63%) |
| Reduced likelihood of damaging an important structure I am unable to see while firing | 4 (50%) |

*Consensus achieved

## DISCUSSION

The goal of an endoscopic linear stapling device is to form an accurate, consistent, and reproducible staple line during thoracic surgery. This study found that instability may lead to a number of negative outcomes. Each tissue type is affected differently by the movement of the distal tip of the device during thoracic surgery. Additionally, endoscopic linear stapling device stability is seen as even more important clinically in VATS compared to open thoracic surgery, which may be attributed to the need to insert multiple instruments into a minor incision, resulting in a small margin for error. Ultimately, an improvement in the stability of an endoscopic linear stapling device is unanimously perceived as having a positive clinical effect on surgical outcomes.

There are several limitations associated with this study. First, the findings may reflect inherent variability of the panelists with respect to their education and clinical experience of thoracic surgery. The opinions and responses expressed by these panelists do not necessarily reflect the position of other surgeons within their country or hospital, and these results may not be applicable to all settings. Moreover, the small number of panelists (n=8) may have resulted in agreement that is less generalizable compared to agreement that would have been achieved with a larger group. Additionally, the consensus formed in this study is based on survey responses, and does not incorporate real-world outcomes in determining the importance of tip stability. Although the panel achieved consensus on several items, this study is also limited because the use of anonymous surveys did not allow for meaningful panel dialogue and deliberation that could have occurred if the panelists were convened for a face-to-face meeting, which could have helped to facilitate consensus.

Although there are no current guidelines addressing the importance of device selection in thoracic surgery, these findings should not replace evidence-based guidance and professional society guidelines. The threshold values used to define consensus were arbitrary, but reflect values found in the literature.[11]

## CONCLUSION

Improved endoscopic linear stapling device stability is a critical component of thoracic surgery procedures that is likely to result in more positive surgical outcomes when compared to a device with greater instability. Because of the sequelae caused by an unstable tip, careful consideration should be taken to determine which endoscopic linear stapling device is selected for thoracic surgery procedures.

## CONFLICT OF INTEREST DECLARATION

Dr. Miller reports grants from Ethicon/Johnson & Johnson, during the conduct of the study. Dr. Gonzalez-Rivas reports grants from Ethicon/Johnson & Johnson, during the conduct of the study. Dr. Meyer reports grants from Ethicon/Johnson & Johnson, during the conduct of the study. Dr. Clark reports grants from Ethicon/Johnson & Johnson, during the conduct of the study. Dr. Kohno reports grants from EthiconJohnson & Johnson, during the conduct of the study.

## Figures and Tables

**Figure:** Supplementary Content

